# Supplementary figures and images for: Tracking Replicating HPV Genomes in Proliferating Keratinocytes
Source: bioRxiv. 2025 Mar 19:2025.03.18.644043. Preprint. [Version 1] doi: 10.1101/2025.03.18.644043 (PMC11956994; doi:10.1101/2025.03.18.644043)

Supplemental figure 1

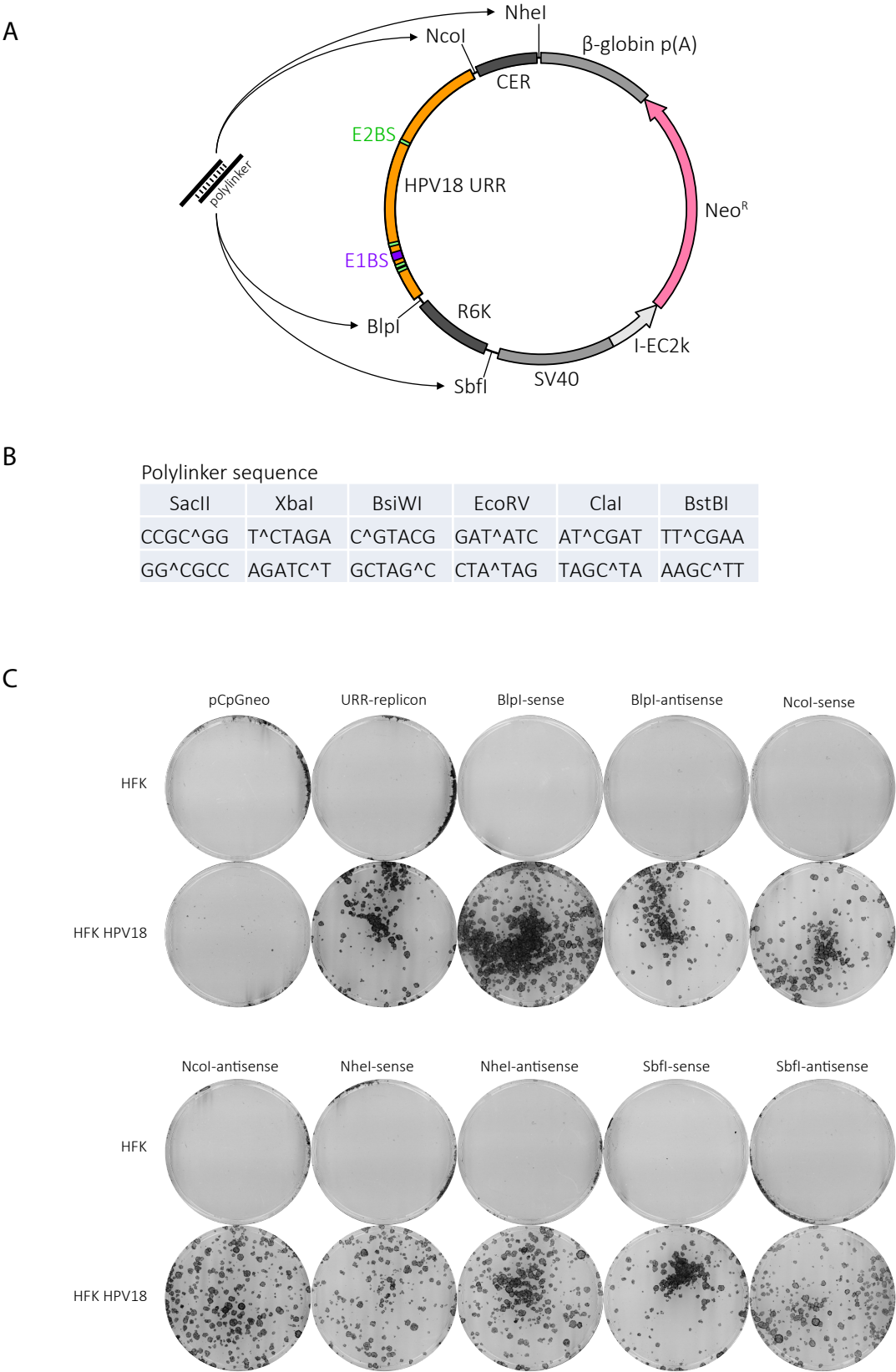

A

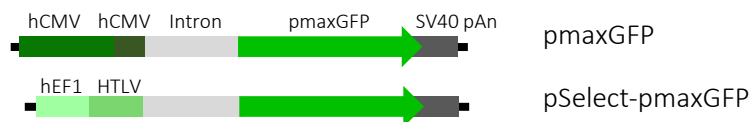

B

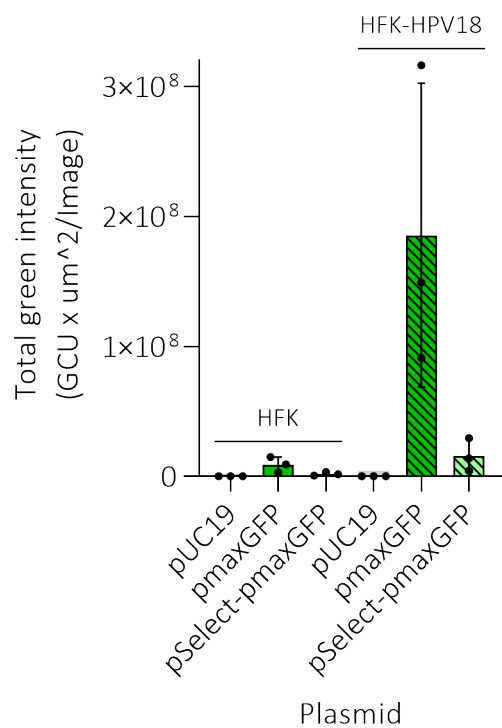

C

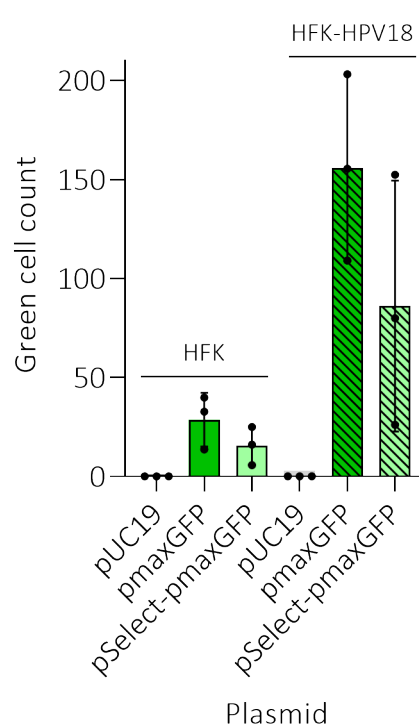

A

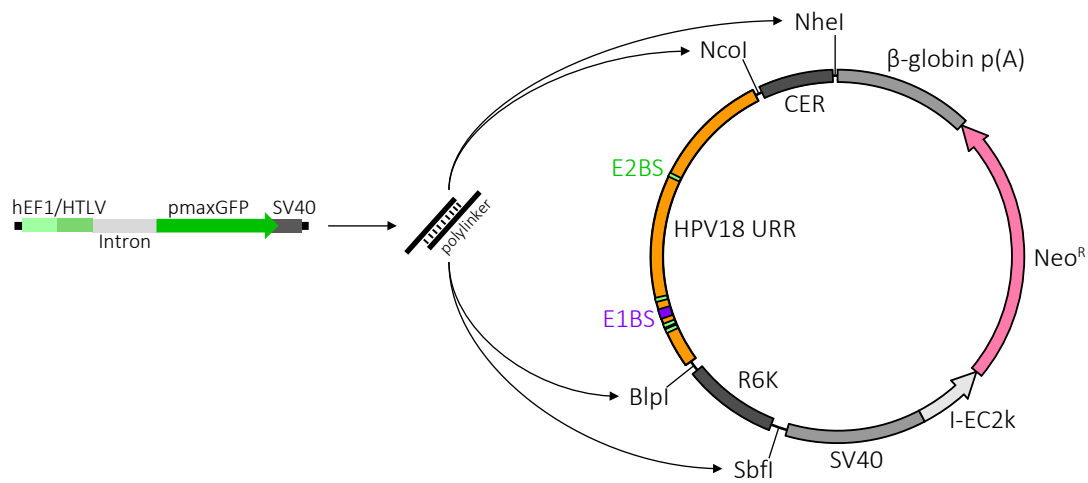

B

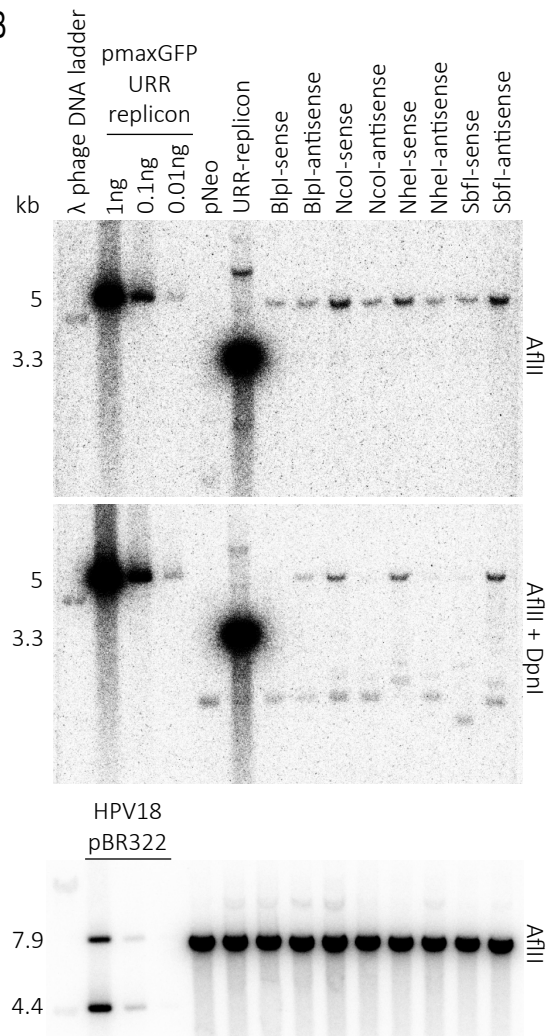

C

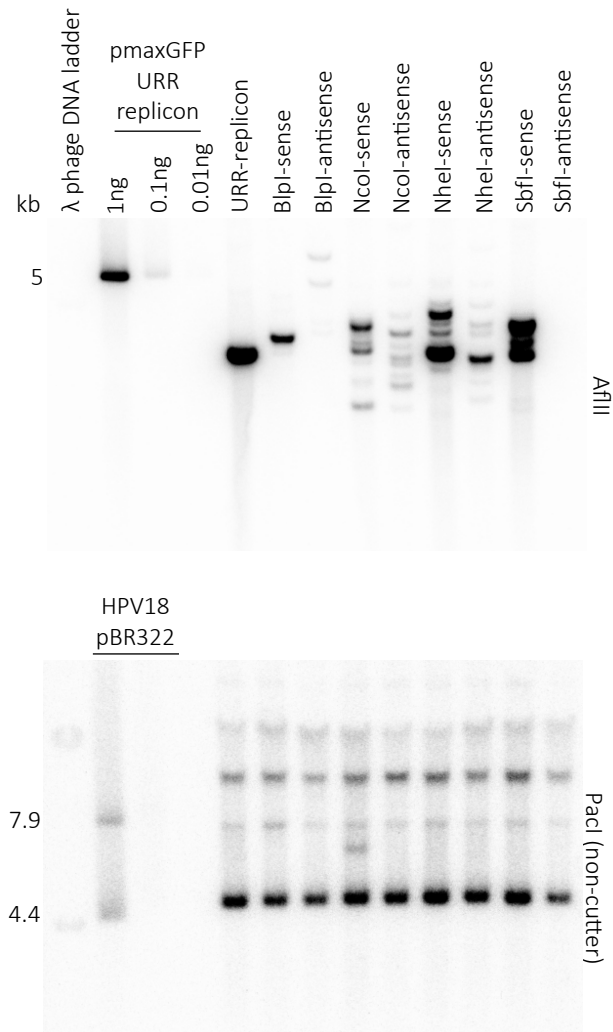

Supplement: Supplement 2 — Supplemental Figure 1. Optimization of the URR-Replicon Expression Vector A polylinker was inserted into four positions in the URR-replicon (in the sense and antisense orientation) between functional elements. Thereafter, each plasmid was electroporated into HFK, or HFK-HPV18 cells to assess their ability to establish as stable URR-replicons. This resulted in stable neomycin-resistant colonies for all plasmids, but only in the presence of HPV18. pCGneo, a neomycin resistance plasmid, does not contain the HPV18 URR and therefore cannot replicate. NB: Blp sense has Increased colonies due to seeding error. Supplemental Figure 2. Developing an optimal expression cassette (A) fluorescent protein gene expression cassettes pmaxGFP and pSelect-pmaxGFP. The plasmids shown were transfected into HPV negative or HPV18 containing HFKs and imaged in a IncuCyte scanner. (B) Total integrated fluorescent intensity at 48 hours post-transfection (n=3, error bars=SD). (C) The green cell count at 48 hours post-transfection (n=3, error bars=SD). Supplemental Figure 3. Replication of the pmaxGFP-URR-replicons in primary HFKs. (A) Map of the URR replicon and cloning positions for pMAX-GFP cassettes. (B) Southern blot assay. The eight pmaxGFP-URR-replicons and pCGneo (pNeo) plasmid were electroporated into HFK-HPV18 cells. DNA was harvested after six days; the cellular DNA in the top panel was digested with AflII and in the middle panel with AflII and DpnI to linearize and detect DpnI resistant replicated DNA. Size/copy number markers are loaded on the left. The top and middle membranes were probed with a URR-deleted replicon, and the bottom with the HPV18 genome. The endogenous HPV18 genomes were maintained at several-hundred copies per cell. (C) The transfected cells were grown with G418 selection for ~4 weeks and genomic DNA isolated The top panel shows a Southern blot with DNA cleaved with AflII to linearize the replicon, and the bottom panel with PacI (a non-cutter) to determine the ex [file media-2.pdf]
